# Evaluation of the relation between the maxillary sinus and the posterior teeth using digital panoramic radiography

**DOI:** 10.25122/jml-2023-0105

**Published:** 2023-08

**Authors:** Hawraa Noori Atallah, Marwah Safaa Ali, Hussein Jameel Abd Noor, Suha Mohammad Sami, Julfikar Haider

**Affiliations:** 1 Department of Oral and Maxillofacial Surgery, University of Kufa, Najaf, Iraq; 2 Department of Engineering, Manchester Metropolitan University, Manchester, United Kingdom

**Keywords:** panoramic radiography, floor of maxillary sinus, upper posterior teeth, MSF: Maxillary Sinus Floor, MS: Maxillary Sinus, Co: Root in contact with sinus floor, Is: Root inside the sinus, Os: Root outside the sinus

## Abstract

This research aimed to determine the relationship between the maxillary posterior teeth and maxillary sinus floor (MSF), as well as the impact of nearby tooth loss on the space between MSF and posterior maxillary roots. A number of 120 digital panoramic radiographs were obtained from the archives of several clinics in Al-Najaf, Iraq, with the overall teeth examined in these radiographs including 236 of the 1^st^ premolars, 227 of the 2^nd^ premolars, 227 of the 1^st^ molars, and 231 of the 2^nd^ molars, from the right and left sides. The distances between the apices of the teeth and the maxillary sinus were determined. There are three categories of relationships between upper posterior teeth roots and MSF. These include type Os (root apex exists below or outside MSF), Type Co (root apex in contact with the MSF), and Type Is (root apex above or inside MSF). Type Os is the most encountered among premolars, Type Co is mostly encountered among the 2^nd^ molars, and Type Is, in the 1^st^ and 2^nd^ molars. The study finds no correlation between age, gender, and the distribution of maxillary posterior tooth roots attached to the MSF. The first premolars were the furthest from MS, while the first molars were the closest. The most frequent link between maxillary molar roots and the MS was the Co-relation for the 2^nd^ maxillary molar and the Is relation for the 1^st^ maxillary molar. There is a non-significant decrease in the distance between the apices of the 1^st^ maxillary premolar, 2^nd^ maxillary premolar, and 1^st^ maxillary molar and the MS before and after extraction.

## INTRODUCTION

The intimate relationship between the roots of posterior teeth and the maxillary sinus (MS) is one of the causes of maxillary sinus complications after dental treatment. The maxillary sinus is a pyramidal-shaped cavity in the body of the maxilla, with the lateral wall of the nose forming the sinus base and the tip of the pyramid extending to the zygomatic process of the maxillary bone. The alveolar process of the maxilla makes the floor of the MS, which is located five millimeters below the nasal floor at the age of 20 [1-2]. Surgeons must have in-depth knowledge of the vast array of variations in the maxillary sinus prior to surgical procedures such as maxillary sinus elevation or dental implant placement [3-4]. One of the anatomic variances is pneumatization, a physiologic process that increases the capacity of the paranasal sinuses during the growing period [5]. Extraction of the posterior maxillary teeth can have an impact on the maxillary sinus pneumatization, particularly the extraction of several neighboring teeth that have apices projected into the MS [2, 6]. The intimate association between the posterior teeth roots and the maxillary sinus can cause issues during the treatment of the maxillary sinus (e.g., extraction), which can result in the establishment of oroantral communication. The root canal treatment method can induce maxillary sinus perforation, allowing dental material to enter the maxillary sinus [7]. Understanding the anatomical link between the MS and the apex of the posterior maxillary teeth is crucial for minimizing or avoiding complications following extraction or root canal therapy [8-9]. The maxillary sinus is often well visible on panoramic radiographs [10]. Panoramic radiography is a traditional imaging tool for examining the tight contact between the roots of the upper posterior teeth and the MS [11]. The aim of this study is to assess the connection between the MS and the upper posterior teeth with the use of digital panoramic radiography.

## MATERIAL AND METHODS

### Panoramic images collection

From November 2021 to January 2022, a total of 120 radiographs (with the overall teeth examined in these radiographs) including 236 of the 1^st^ premolars, 227 of the 2^nd^ premolars, 231 of the 1^st^ molars, and 236 of the 2^nd^ molars from the right and left sides were obtained from the archives of digital panoramic radiography in several clinics in Al-Najaf, Iraq. The relation between the apices of all the mentioned teeth and the MS was assessed by making a radiographic report describing the relation and using a digital ruler included in the program of taking the radiograph. The impact of nearby tooth loss on the relation between MSF and posterior maxillary roots was evaluated by measuring the distance between the root apex of the tooth which is just adjacent to the extracted tooth, to the floor of the sinus.

The inclusion criteria were:

- high-quality and ideal panoramic radiographs;
- fully formed upper posterior permanent teeth where the root apex is closed and without any pathological condition;
- healthy maxillary sinus condition without any pathology.

The exclusion criteria were cysts, periapical abscesses and granuloma, hypercementosis, periodontal disorder, and sinus inflammation.

### Panoramic images evaluation

**Figure 1:**
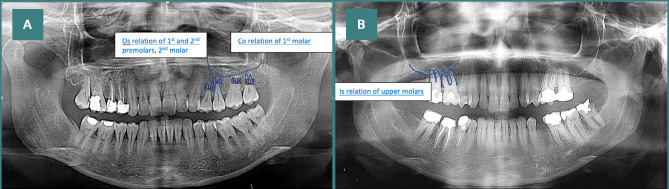
Examples of (A) Co and Os and (B) Is relations of teeth to the MS showing in panoramic radiographs

All images were analyzed by a radiologist with extensive expertise. An example of the panoramic image is shown in Figure 1. The relationship type between the roots of the upper posterior teeth and the MSF was reported as:

- Type (Os), where the root apex exists outside the MSF
- Type (Co), where the root apex is in contact with the floor of the sinus
- Type (Is), where the root apex exists inside the MSF.

The impact of nearby tooth loss on the space between MSF and posterior maxillary roots was evaluated by measuring the distance between the root apex of the tooth which is just adjacent to the extracted tooth, to the floor of the sinus. Using the digital ruler included in the panoramic imaging software (iRYS viewer), the distances from the teeth apices to the MSF were also measured. If a tooth was multirooted, the closest root to the MSF was evaluated.

### Statistical analysis

Statistical analysis was carried out using version 26 of SPSS. Categorical variables were presented as frequencies and percentages. As necessary, Chi-square and Fisher's exact tests evaluated the correlations between the categorical variables. Mann-Witney U test was used to make a comparison in the difference between the presence or absence of a neighboring tooth. Results were considered significant at p≤0.05.

## RESULTS

Table 1 depicts the distribution of the relationship of posterior maxillary root apices with the MSF type Os being the most common relationship between 1^st^ premolar root apices and the MSF (87%), followed by the relationship between the 2^nd^ premolar and MSF (57.7%). This type of relationship is less encountered between molars and the maxillary sinus floor (24.2% in the case of the first molar, 15.6% in the case of the second molar). Type Co was the most prevalent in the second molars (46.3%), followed by the first molars (34.8%), the second premolars (33.5%), and the first premolars (11%). Type Is relationship is the most prevalent in the first and second molars (41% and 38.1%, respectively).

**Table 1:** Distribution of the relation between root apex of upper posterior teeth and floor of the maxillary sinus

	Os No (%)	Co No (%)	Is No (%)	Total
1^st^ Premolar	206 (87.3%)	26 (11%)	4 (1.7%)	236 (100%)
2^nd^ Premolar	131 (57.7%)	76 (33.5%)	20 (8.8%)	227 (100%)
1^st^ molar	55 (24.2 %)	79 (34.8%)	93 (41%)	227 (100 %)
2^nd^ molar	36 (15.6 %)	107 (46.3%)	88 (38.1%)	231 (100 %)

Co: root in contact with sinus floor; Is: inside the sinus; Os: root outside the sinus

As showcased in Table 2, there is no effect of age and gender on the distribution of the relations of upper posterior teeth roots to the MSF.

**Table 2:** Age and gender associated with the distribution of the relation of root apex of posterior teeth and MSF

Female		Gender		Total	p	Age		Total	p
		Female	Male			<35	≥35		
First premolar	Co	19 (73.1%)	7 (26.9%)	26 (100%)	0.3	17 (65.4%)	9 (34.6%)	26 (100%)	0.4
	Is	4 (100.0%)	0 (0.0%)	4 (100%)		2 (50.0%)	2 (50.0%)	4 (100%)	
	Os	133 (64.6%)	73 (35.4%)	206 (100%)		106 (51.5%)	100 (48.5%)	206 (100%)	
Total		156 (66.1%)	80 (33.9%)	236 (100%)		125 (53%)	111 (47%)	236 (100%)	
Second premolar	Co	46 (60.5%)	30 (39.5%)	76 (100%)	0.1	41 (53.9%)	35 (46.1%)	76 (100%)	0.9
	Is	17 (85.0%)	3 (15.0%)	20 (100%)		11 (55.0%)	9 (45.0%)	20 (100%)	
	Os	86 (65.6%)	45 (34.4%)	131(100%)		71 (54.2%)	60 (45.8%)	131 (100%)	
Total		149 (65.6%)	78(34.4%)	227 (100%)		123 (54.2%)	104 (45.8%)	227 (100%)	
First Molar	Co	46 (58.2%)	33 (41.8%)	79 (100%)	0.1	40 (50.6%)	39 (49.4%)	79 (100%)	0.6
	Is	65 (69.9%)	28 (30.1%)	93 (100%)		52 (55.9%)	41 (44.1%)	93 (100%)	
	Os	40 (72.7%)	15 (27.3%)	55 (100%)		32 (58.2%)	23 (41.8%)	55 (100%)	
Total		151 (66.5%)	76 (33.5%)	227 (100%)		124 (54.6%)	103 (45.4%)	227 (100%)	
Second Molar	Co	73 (68.2%)	34 (31.8%)	107 (100%)	0.5	55 (51.4%)	52 (48.6%)	107 (100%)	0.4
	Is	55 (62.5%)	33 (37.5%)	88 (100%)		45 (51.1%)	43 (48.9%)	88 (100%)	
	Os	26 (72.2%)	10 (27.8%)	36 (100%)		23 (63.9%)	13 (36.1%)	36 (100%)	
Total		154 (66.7%)	77 (33.3%)	231 (100%)		123 (53.2%)	108 (46.8%)	231 (100%)	

The effect of the extraction on the adjacent site is shown in Table 3. There is a decrease in the distance between the apices of (maxillary first premolar, maxillary second premolar, and maxillary first molar) and MS, leading to maxillary sinus pneumatization after extraction. Still, the difference in distances before and after extraction does not reach the significance level.

**Table 3:** Comparison of the vertical distances of the root apices to the sinus floor according to the presence or absence of adjacent teeth

Maxillary first premolar	Presence of adjacent 2^nd^ premolar Median (IQR)*	Absence of adjacent 2^nd^ premolar Median (IQR)	p-value
4.4 (2.9)	2.7 (3.1)	0.06
**Maxillary second premolar**	Presence of adjacent 1^st^ premolar Median (IQR)*	Absence of adjacent 1^st^ premolar Median (IQR)	p-value
	2 (3.1)	0.15 (3)	0.2
**Maxillary first molar**	Presence of adjacent 2^nd^ molar Median (IQR)*	Absence of adjacent 2^nd^ molar Median (IQR)	p-value
	0 (1.7)	-1.6 (2.8)	0.07
**Maxillary second molar**	Presence of adjacent 1^st^ molar Median (IQR)*	Absence of adjacent 1^st^ molar Median (IQR)	p-value
	0 (1)	0 (1.05)	0.2

* Interquartile range

## DISCUSSION

The rough anatomical proximity of the root apices of upper posterior teeth with the MS results in many difficulties throughout dental treatment. Due to the risk of pneumatization that occurs after extraction, which minimizes the quantity of bone available for implant placement or denture construction, clinicians performing pre-prosthetic and pre-implant surgical operations in the posterior part of the maxillary jaw must know the number of teeth roots that emerge in the maxillary sinus [12]. In spite of the fact that the cone-beam computed tomography system gives more accurate details, panoramic radiography was used in this study due to a lack of cases in the archive of the cone-beam computed tomography system in several clinics and due to cost and radiation dose that limit its use Additionally, a panoramic radiograph used by the majority of dentists to evaluate the Orofacial complex as it gives an adequate view of jaws, sinuses, and teeth. This study examined the relations of the upper posterior root apices with the MSF in Iraqi people at Al-Najaf city. Type Os was the most common relationship between the maxillary first premolars to the sinus floor, which is consistent with the research of Abdulwahed *et al*. (2023), Pagin *et al*. (2013), and Tian *et al*. (2016) [12-14]. For maxillary second premolars, the frequency of Os relationship decreased (57.7%) and the frequency of Co and Is relationships increased when compared with the maxillary first premolars. This indicated that the second maxillary premolars had a closer connection with the maxillary sinus floor, which is in agreement with a study conducted in 2018 by Gu *et al*. [8]. Based on the results of this study, the type Is relationship occurs more commonly in the maxillary first molars (41%), followed by the maxillary second molars (38.1%), while the type Co relationship occurs more frequently in the maxillary second molars (46.3%) and first molars (34.8%). This suggests that dentists should become more attentive to these locations during dental procedures, as perforation of the maxillary sinus floor may occur. This outcome is consistent with Arabion *et al*. and Pertiwi *et al*. [15-16]. Similar to the research of Gu *et al*. [8] and Pei *et al*. [17], the two sides were studied together, revealing no statistically significant difference between the right and left sides. Age and gender showed no significant impact on the results A possible explanation for the absence of any obvious or consistent effect of age or gender is the restricted sample size. This finding is consistent with Von Arx *et al*. [18]. The distance between MSF and root apices of upper posterior teeth can be altered by tooth extraction. This study measured the distance between the apices of upper posterior teeth and MSF with the presence and absence of adjacent teeth for each tooth and found that the distance decreased when the adjacent tooth was extracted. Hence, expansion in the maxillary sinus occurred, although the outcomes were not statistically significant. Von Arx *et al*. [18] and Gu *et al*. [8] reached a similar conclusion about the absence of a substantial impact of extraction.

## CONCLUSION

This retrospective study showcased several conclusions. Firstly, it was revealed that the first premolar had the longest distance from the sinus floor, and the first molars had the shortest distance. Furthermore, the Co relationship was the most common for the maxillary second molar, while the Is relationship was more prevalent for the 2^nd^ molars. Age and gender had no significant effect on the results. Lastly, a decrease in the distance between maxillary premolars root apices and maxillary sinus, or the maxillary molars and maxillary sinus after extraction of the adjacent tooth was highlighted, yet the findings were not significant.
